# Author Correction: Azobenzene-based optoelectronic transistors for neurohybrid building blocks

**DOI:** 10.1038/s41467-023-43621-4

**Published:** 2024-01-24

**Authors:** Federica Corrado, Ugo Bruno, Mirko Prato, Antonio Carella, Valeria Criscuolo, Arianna Massaro, Michele Pavone, Ana B. Muñoz-García, Stiven Forti, Camilla Coletti, Ottavia Bettucci, Francesca Santoro

**Affiliations:** 1 Institute of Biological Information Processing IBI-3 Bioelectronics, Forschungszentrum Juelich, 52428 Juelich, Germany; 2 Neuroelectronic Interfaces, Faculty of Electrical Engineering and IT, RWTH Aachen, 52074 Aachen, Germany; 3 Tissue Electronics, Center fo Advanced Biomaterials for Healthcare, Istituto Italiano di Tecnologia, 80125 Naples, Italy; 4 Dipartimento di Ingegneria Chimica, dei Materiali e della Produzione Industriale, Università degli Studi di Napoli Federico II, 80125 Naples, Italy; 5 Materials Characterization Facility, Istituto Italiano di Tecnologia, 16163 Genoa, Italy; 6 Dipartimento di Scienze Chimiche, Università degli Studi di Napoli “Federico II”, Complesso Universitario Monte S. Angelo, 80126 Naples, Italy; 7 Dipartimento di Fisica “E. Pancini”, Università degli Studi di Napoli “Federico II”, Complesso Universitario Monte S. Angelo, 80126 Naples, Italy; 8 Center for Nanotechnology Innovation, Istituto Italiano di Tecnologia, 56127 Pisa, Italy; 9 Present Address: Department of Materials Science and Milano-Bicocca Solar Energy Research Center – MIB-Solar, University of Milano-Bicocca, 20125 Milano, Italy

**Keywords:** Electronic devices, Electrical and electronic engineering, Biomedical engineering, Sensors and biosensors

Correction to: *Nature Communications* 10.1038/s41467-023-41083-2, published online 02 November 2023

The original version of this Article contained an error in Fig. 2g-ii, in which the entire graph is missing.

The correct version of Fig. 2 is:


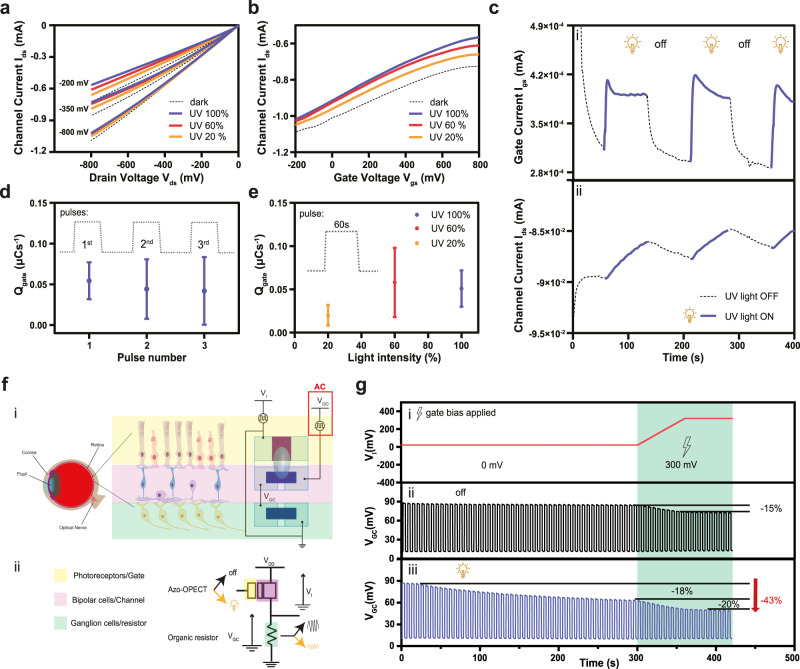


which replaces the previous incorrect version:


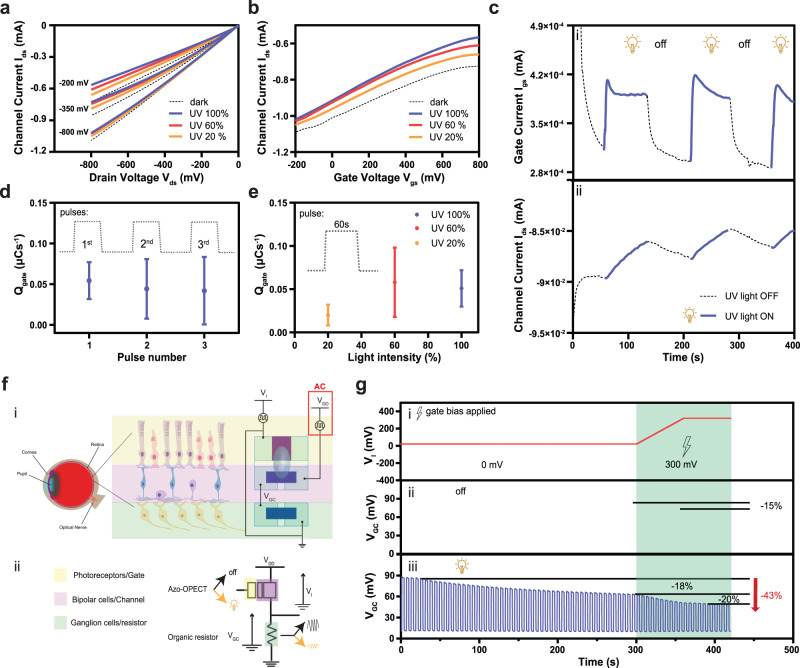


This has been corrected in both the PDF and HTML versions of the Article.

