# Supplementary material for: Azobenzene-based optoelectronic transistors for neurohybrid building blocks
Source: Nat Commun. 2023 Nov 2;14:6760. doi: 10.1038/s41467-023-41083-2 (PMC10622443; doi:10.1038/s41467-023-41083-2)
Supplement: Supplementary file 1 — Supplementary Information [file 41467_2023_41083_MOESM1_ESM.pdf]

Supplementary Figures

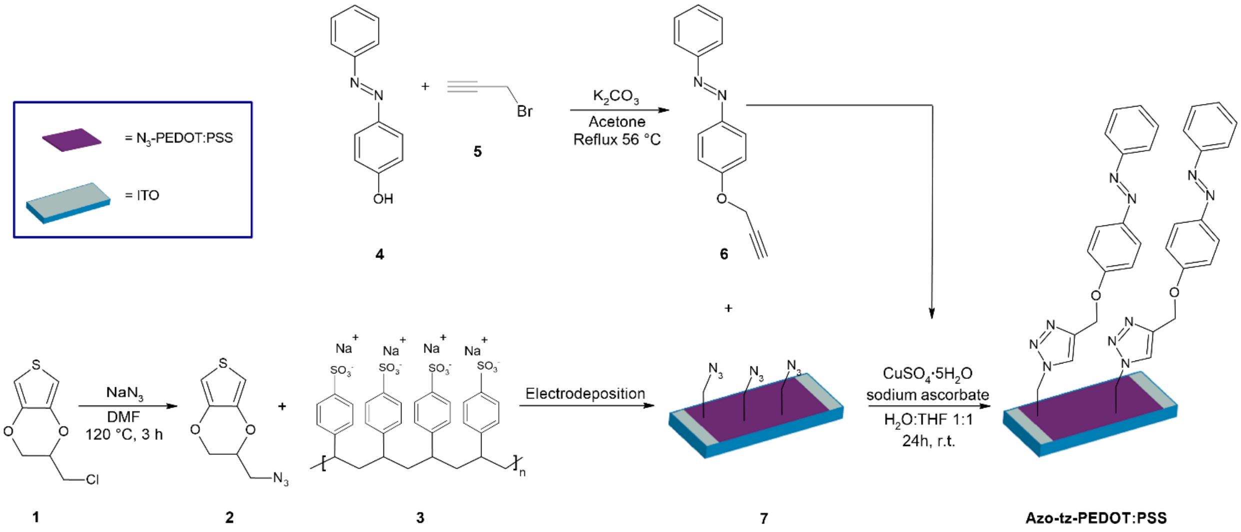

Supplementary Figure 1. Synthetic route of azo-tz-PEDOT:PSS. Synthesis of N_3-EDOT (2) and azo-alkyne (6) monomers[2,3]; electrodeposition of N_3-PEDOT:PSS (7) on an ITO substrate and post-functionalization of the electrodeposited PEDOT film through click chemistry. To obtain N_3-PEDOT:PSS, a monomer precursor, N_3-EDOT (2), was synthesized as shown in the Methods[1]. N_3-EDOT (2) was then electropolymerized by cyclic voltammetry (CV) on conductive ITO-coated glass. The electropolymerized N_3-PEDOT film was post-functionalized *via Cu(I)-catalyzed azide-alkyne Huisgen [3+2] cycloaddition* in the presence of azo-alkyne (6) (Methods) following a reported procedure to obtain azo-tz-PEDOT:PSS[3].

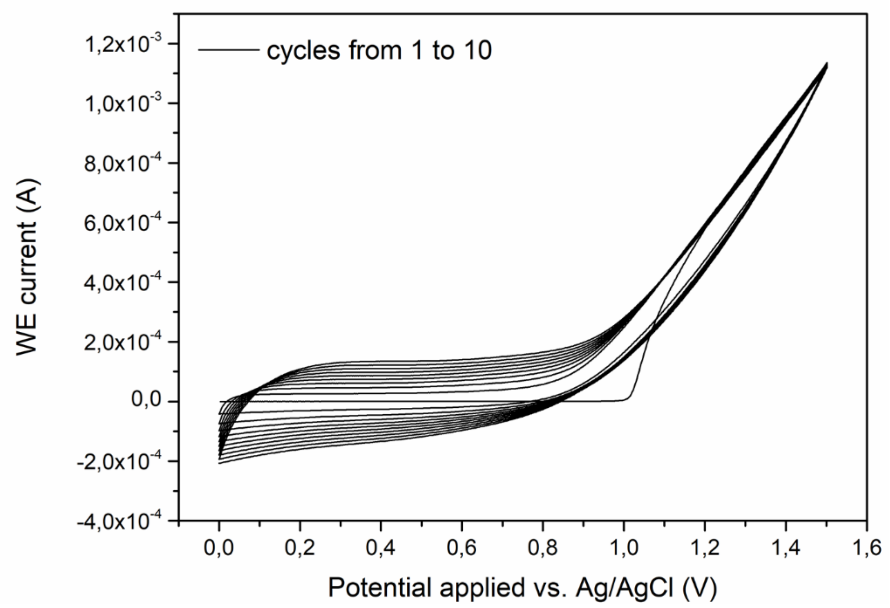

Supplementary Figure 2. N_3-PEDOT:PSS electropolymerization by cyclic voltammetry. CV curves obtained during the electropolymerization from a N_3-EDOT aqueous dispersion. N_3-EDOT was electropolymerized by cyclic voltammetry (CV) in the potential range between 0 V and 1.5 V using a three-electrode setup consisting of an ITO electrode (working electrode, WE), a platinum wire (counter electrode, CE), and an Ag/AgCl electrode (reference electrode, RE) (Methods)[1]. The voltammograms obtained during the electrodeposition of the EDOT-N_3 monomer, reported in Figure 2, show a rapid film growth process. During the first cycle, the anodic current density of N_3-EDOT starts to rapidly increase from 0 A to 1.1×10^{-3} A. Here, continuous increases in the cathodic and anodic currents over the CV cycles were observed, indicating successful electrodeposition of the conductive polymer on the WE, allowing us to obtain a homogeneous polymeric film of N_3-PEDOT:PSS in a single-step reaction[3].

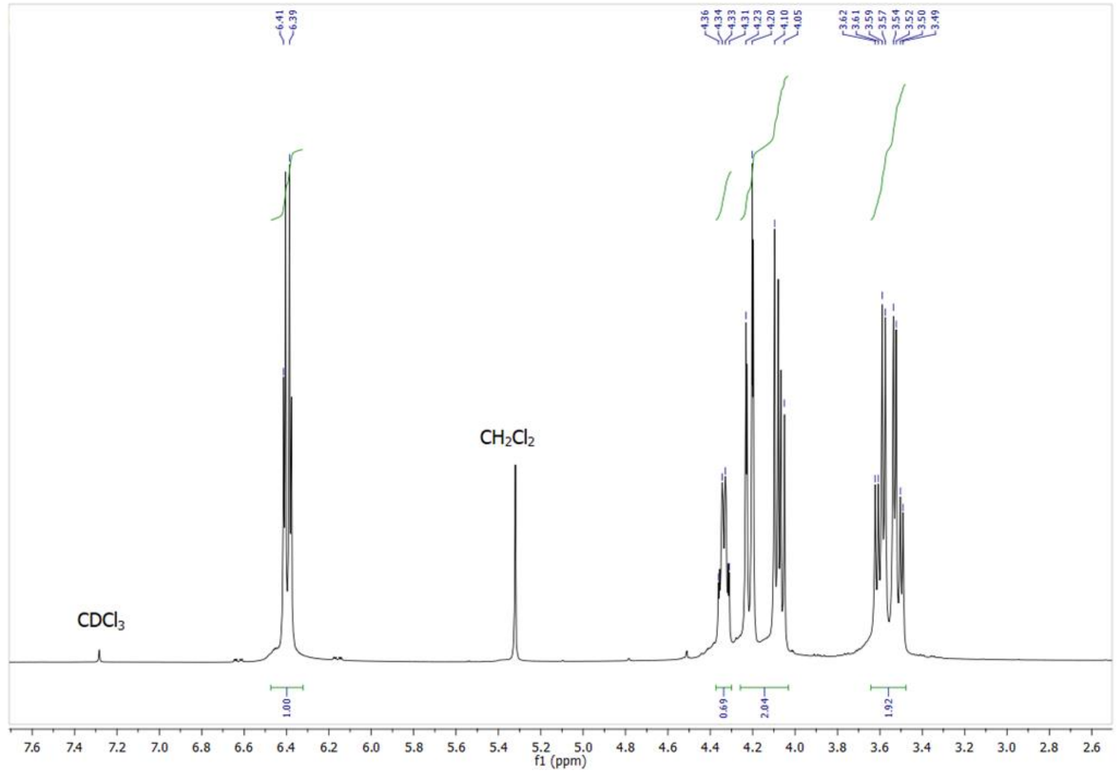

Supplementary Figure 3. ^{1}H NMR spectrum of N_3-EDOT. ^{1}H NMR spectrum in $CDCl_3$ (400 MHz) of N_3-EDOT (2). ^{1}H NMR ($CDCl_3$, 400 MHz, δH, ppm): 3.56 (ddd, 2H, -CH_2-N_3); 4.07, 4.21 (dd, 2H, -O-CH_2), 4.35 (m, 1H, -O-CH), 6.40 (dd, 2H, -S=CH). The ^{1}H NMR spectrum of N_3-EDOT (2) showed a diagnostic signal at 3.56 ppm, attributed to the hydrogens of the methylene linked to the azide functionality (–CH_2–N_3), while two signals at 4.07 ppm and 4.21 ppm were attributed to the hydrogens of methylene linked to the oxygen (–O–CH_2). Two other characteristic peaks were observed at 4.35 ppm and 6.40 ppm, attributed to the hydrogen in the –O–CH region and to the hydrogen near the sulfur atom (–S=CH) in the thiophene ring, respectively. All signals observed for (2) agree with the literature data[2].

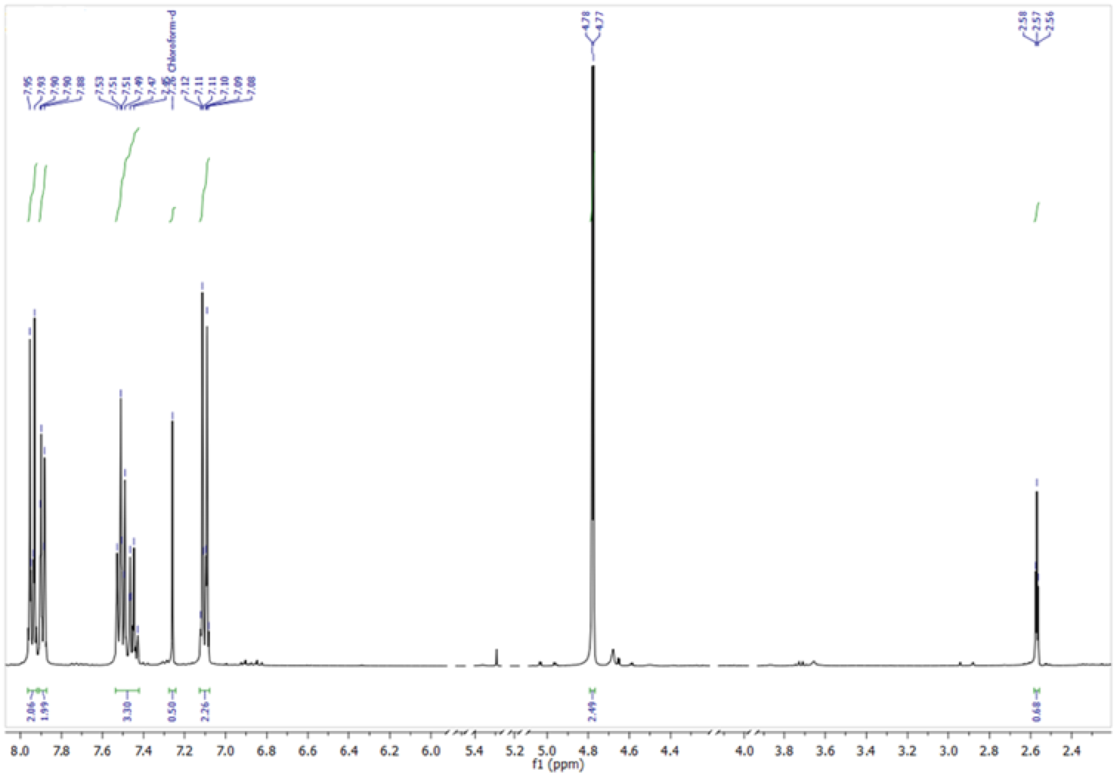

Supplementary Figure 4. ^{1}H NMR spectrum of azo-alkyne. ^{1}H NMR spectrum in $CDCl_3$ (400 MHz) of azo-alkyne (6) ^{1}H NMR ($CDCl_3$, 400 MHz, δH, ppm): 7.94 (m, 2H, H-2), 7.89 (dt, 2H, H-2'), 7.54–7.42 (m, 3H, H-3', H-4'), 7.12 (m, 2H, H-3), 4.78 (d, 2H, CH_2), 2.57 (t, 1H, H-C≡C-). The ^{1}H NMR spectrum of (6) showed two diagnostic signals at 4.78 ppm and at 2.57 ppm attributed to the hydrogens of the methylene –CH_2– and to the hydrogen linked to the terminal alkyne (H–C≡C–), respectively. All signals observed for (6) agree with the literature data[3].

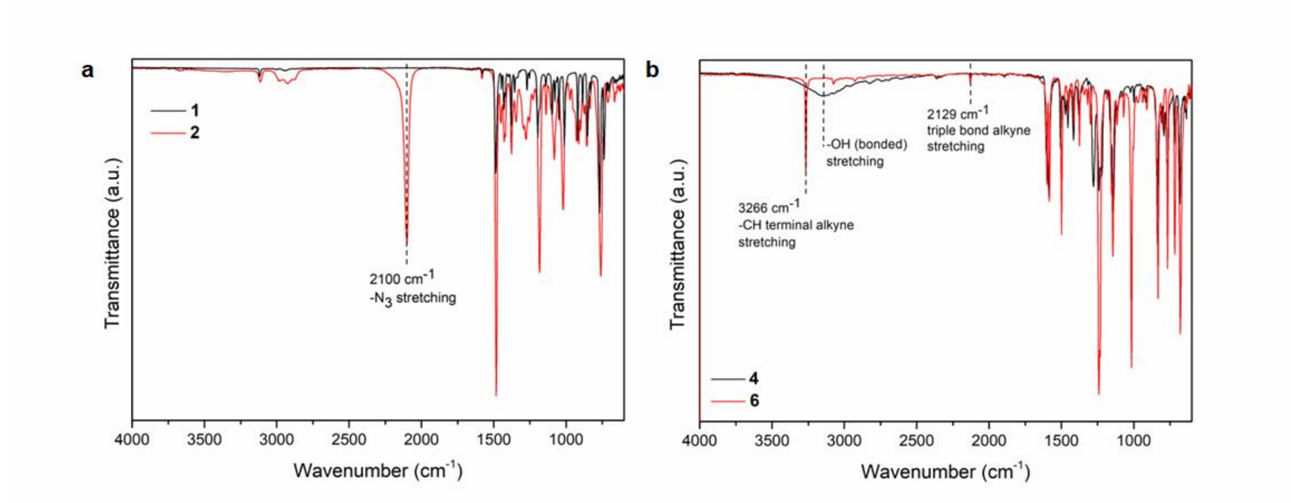

Supplementary Figure 5. FTIR-ATR spectra of precursors. a) FTIR-ATR spectrum of N_3-EDOT (2, red solid line) compared with Cl-EDOT (1, black solid line). b) FTIR-ATR spectrum of the azo-alkyne (6, red solid line) compared to 4-(phenylazo)phenol (4, black solid line). The comparison between the FTIR-ATR spectra of the Cl-EDOT precursor (1) (black solid line) and N_3-EDOT (2) (red dashed line) shows the appearance of a sharp diagnostic peak at 2100 cm^{-1} in the spectrum of (2) corresponding to the azide stretching vibration band and confirming the conversion of product (1) into (2). The reported spectra agree with the literature[1,4]. The FTIR-ATR spectra of the 4-(phenylazo)phenol precursor (4) (black solid line) with the azo-alkyne (6) (red dashed line) show the presence of two peaks at 3266 cm^{-1} and 2129 $cm^{-1,}$ which correspond to the -C-H stretching of the terminal alkyne and the –C≡C– stretching, respectively. Moreover, the absence of the broad signal at 3000 cm^{-1}, corresponding to the -OH stretching vibration band of (4), further confirmed the success of the reaction. These results are in accordance with those reported in the literature[3].

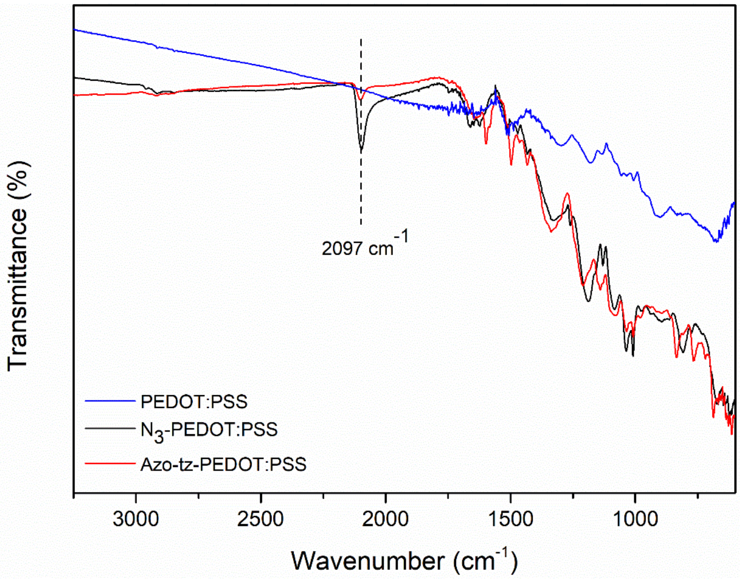

Supplementary Figure 6. Comparison of FTIR-ATR spectra of polymers. FTIR-ATR spectra of PEDOT (blue solid line), N_3-PEDOT:PSS (black solid line), and azo-tz-PEDOT:PSS (red solid line). From the comparison of the FTIR-ATR spectra of PEDOT:PSS (blue solid line), N_3-PEDOT:PSS (black solid line) and azo-tz-PEDOT:PSS (red solid line), a decrease in the azide stretching vibration band at 2097 cm^{-1} was observed, confirming the conversion of the azide functionality into the triazole ring. However, the azide band did not completely disappear, suggesting partial post-functionalization.

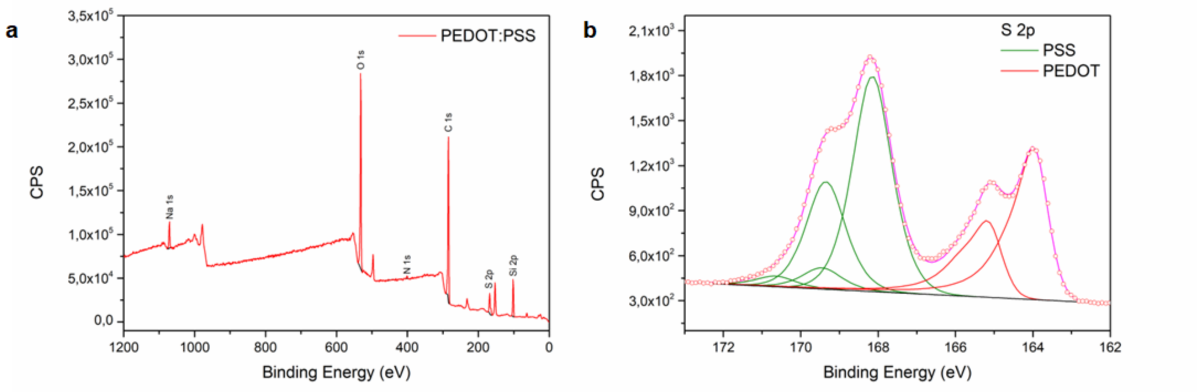

Supplementary Figure 7. XPS analysis of bare PEDOT:PSS. XPS a) *survey spectrum* of PEDOT:PSS and b*)* S 2*p* *core level spectrum* of PEDOT:PSS. The surface elemental composition of the bare PEDOT:PSS electrodeposited film and the amount of entrapped PSS during electrochemical polymerization were investigated through XPS analysis. A preliminary analysis was performed, acquiring a *survey spectrum* of the bare PEDOT:PSS showing all characteristic peaks of the atoms present in the polymer structure: O 1*s* (24,4%), C 1*s* (60,8%), and S 2*p* (4,80%). These results are in agreement with those reported in the literature[5]. To evaluate the amount of entrapped PSS^- during electrochemical polymerization, two different oxidation states of sulfur atoms (S 2*p*) were evaluated through the acquisition of XPS spectra in high-resolution mode (*core level spectra*). A doublet peak at 164.0 eV and 165.1 eV corresponding to the sulfur atom peaks contained in the thiophene ring of the PEDOT backbone was observed[6]. Moreover, an additional doublet peak was noted at 168.0 eV and 169.0 eV, which corresponds to the sulfur atoms present in the PSS moieties entrapped in the polymer film[6]. By considering the peak areas, the PEDOT/PSS ratio was calculated to be 1:2.25, as also previously shown[7].

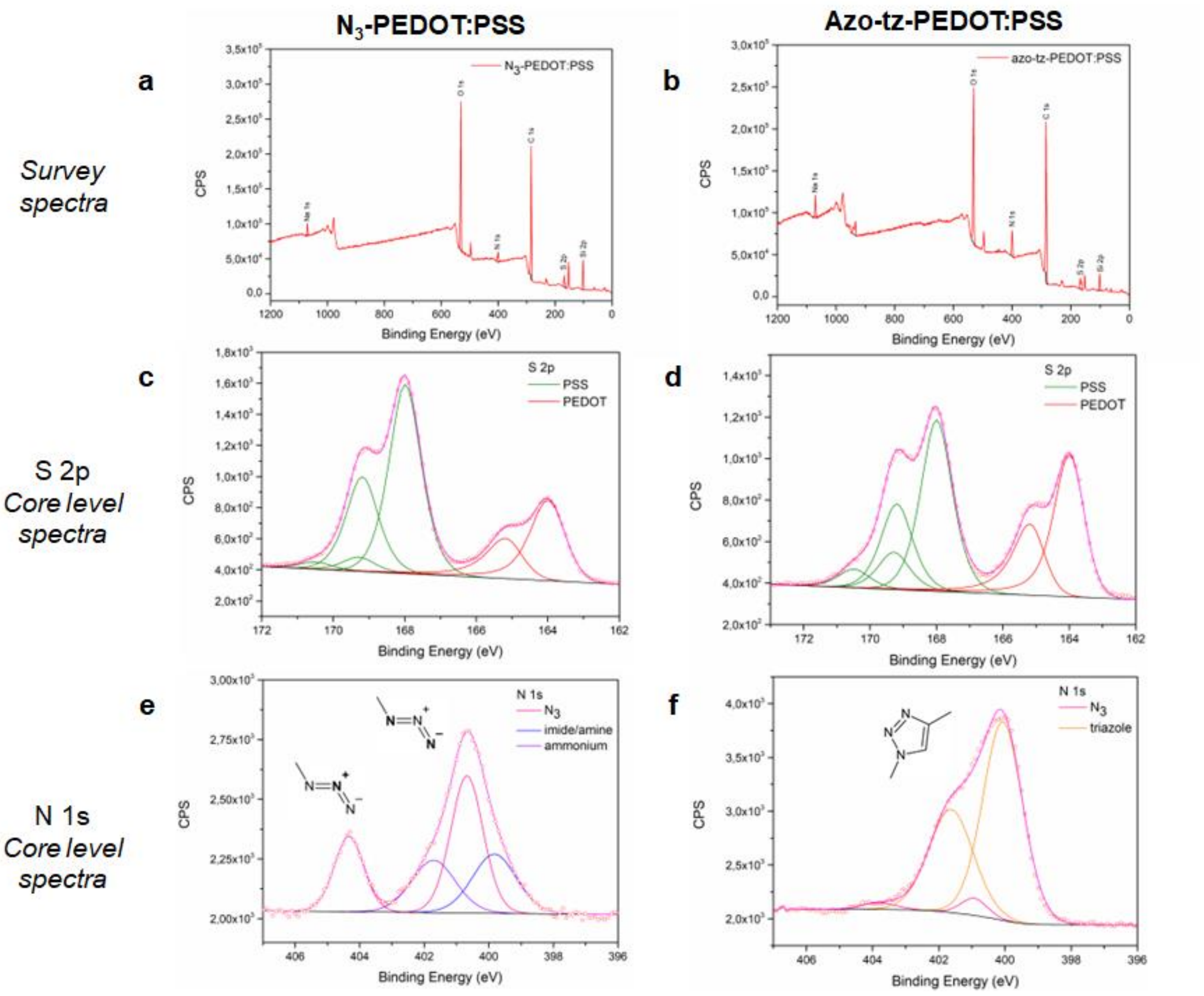

Supplementary Figure 8. XPS analysis of N3-PEDOT:PSS and azo-tz-PEDOT:PSS. a) XPS *survey spectrum* of electrodeposited N_3-PEDOT:PSS on ITO; b) XPS *survey spectrum* of electrodeposited azo-tz-PEDOT:PSS films on ITO; c) XPS *core level spectra* in the S 2*p* region N_3-PEDOT:PSS; d) XPS *core level spectra* of the S 2*p* azo-tz-PEDOT:PSS; e) XPS *core level spectra* in the N 1*s* region of N_3-PEDOT:PSS; f) XPS *core level spectra* in the N 1*s* region of azo-tz-PEDOT:PSS. The surface elemental composition of N_3-PEDOT:PSS was investigated, acquiring XPS *survey spectra* showing all-characteristic peaks of the atoms present in the polymer structure: O 1*s* (20.8%), C 1*s* (62.0%), S 2*p* (3.04%) N 1*s* (3.22%) and a PEDOT/PSS ratio of 1:2.01. All data are in agreement with those found in the literature for bare PEDOT:PSS[8]. The same analysis carried out on azo-tz-PEDOT:PSS showed comparable results in terms of characteristic peaks and percentages of atoms: O 1*s* (18.8%), C 1*s* (66.7%), S 2*p* (2.73%) and N 1*s* (5.07%). Even in this case, the calculated PEDOT/PSS ratio was 1:1.81, in agreement with those reported for both bare PEDOT:PSS and N_3-PEDOT:PSS. Such a result indicates that post functionalization does not affect the PEDOT:PSS backbone. Moreover, to determine changes in the oxidation of nitrogen atoms (N 1*s*) and thus the successful conversion of -N_3 into a triazole ring, high-resolution spectra of nitrogen were acquired. The *core level spectra* in the N 1*s* region of N_3-PEDOT:PSS show the presence of two peaks at 404.3 eV and 400.6 eV corresponding to the

electron-deficient nitrogen (-N=**N**=N) and to the two partially negatively charged nitrogen atoms (-**N**=N=**N**) of the -N_3 group. All data agree with those reported in the literature[8]. Both peaks decrease dramatically in the *core level spectra* of azo-tz-PEDOT:PSS, confirming the disappearance of the -N_3 group after the functionalization reaction. Moreover, a broadening of the peak at 400.6 eV was perfectly fitted by the tabulated peaks of the triazole ring (400.7 eV and 401.7 eV), as reported in the literature[8].

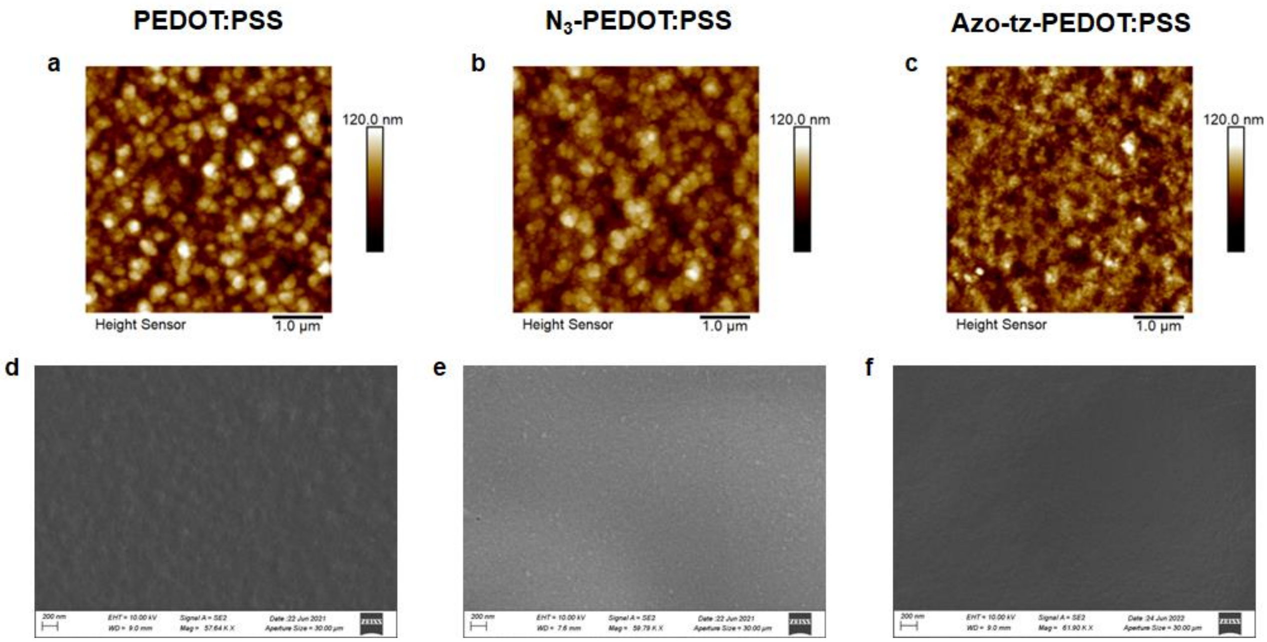

Supplementary Figure 9. Morphology characterization through acquisition of AFM and SEM. AFM images of a) PEDOT:PSS, b) N_3-PEDOT:PSS and c) azo-tz-PEDOT:PSS films. SEM images of d) PEDOT:PSS, e) N_3-PEDOT:PSS and f) azo-tz-PEDOT:PSS films. The morphologies of electrodeposited PEDOT:PSS, N_3-PEDOT:PSS and azo-tz-PEDOT:PSS films were characterized through atomic force microscopy (AFM) and scanning electron microscopy (SEM). The AFM analysis of bare PEDOT:PSS and N_3-PEDOT:PSS shows a globular surface with PSS (dark regions) and PEDOT domains (bright regions) randomly distributed in the polymer matrix, in good agreement with the literature data[2]. The AFM image of azo-tz-PEDOT:PSS does not show significant differences in the topography of the film. Here, PEDOT:PSS, N_3-PEDOT:PSS and azo-tz-PEDOT:PSS have a mean RMS roughness of 15.7 ± 2.0 nm, 10.7 ± 2.0 nm and 10.5 ± 3.1 nm, respectively (N = 3).

Additionally, SEM images of the azo-tz-PEDOT:PSS show a smooth morphology (N = 3) similar to the N_3-PEDOT:PSS precursor and PEDOT:PSS, suggesting that the 'click' reaction only introduces a limited smoothening of the surface.

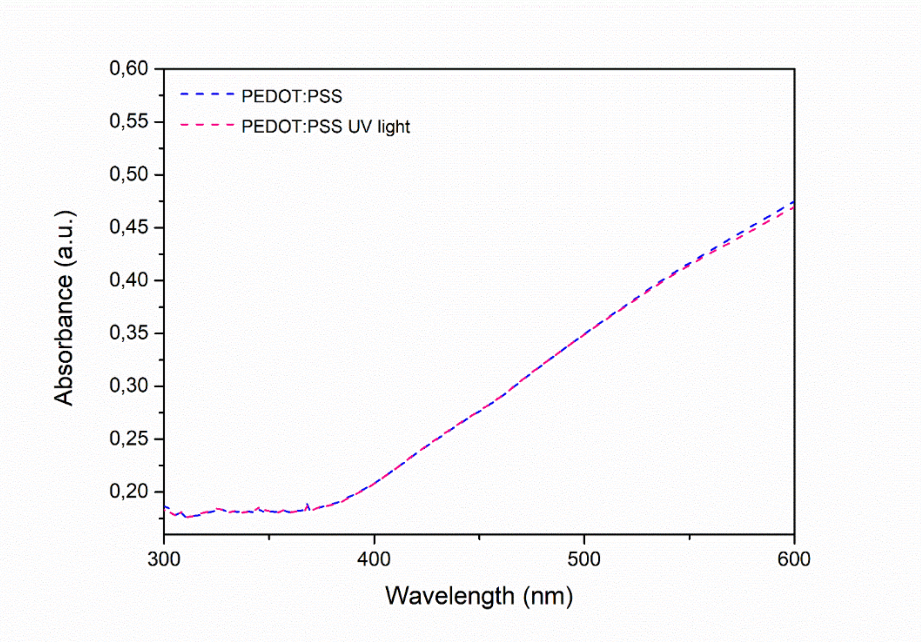

Supplementary Figure 10. UV–visible spectra of PEDOT:PSS. UV–visible spectra of PEDOT:PSS acquired before (blue dashed line) and after (pink dashed line) 5 min of UV irradiation (λ = 365 nm, 6 W). The spectra show no absorption change after light stimulation.

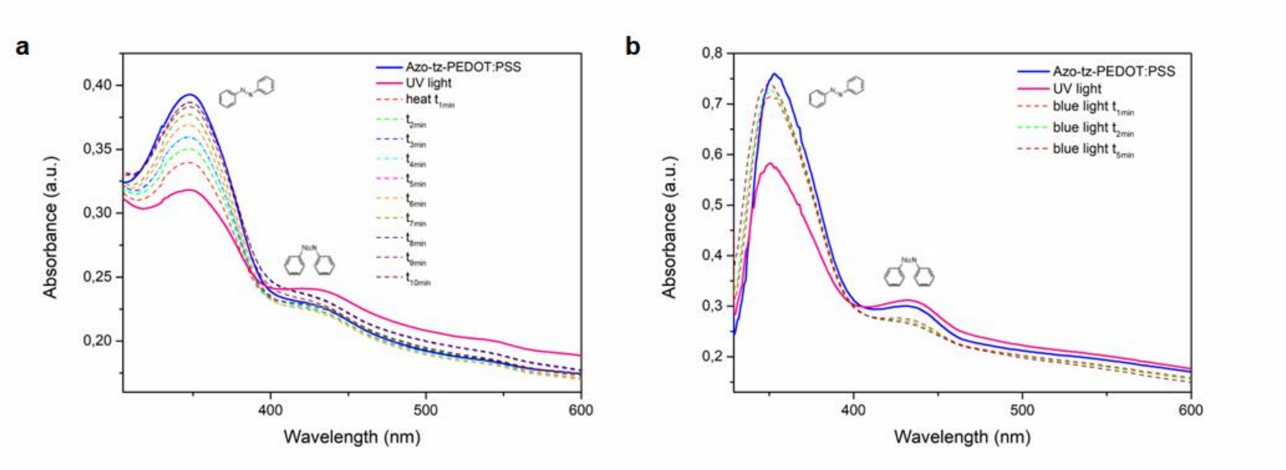

Supplementary Figure 11. UV-visible spectra of azo-tz-PEDOT:PSS. a) UV–visible spectra of azo-tz-PEDOT:PSS acquired before and after 5 minutes of UV irradiation (λ = 365 nm, 6 W) and after 10 minutes of heating at T = 80°C. b) UV–visible spectra of azo-tz-PEDOT:PSS acquired before and after 5 minutes of UV irradiation (λ = 365 nm, 6 W) and after 5 minutes of blue light (λ = 445 nm, 440 mW) irradiation. After UV light stimulation to induce the *trans-cis* photoisomerization of the azobenzenes, *cis-trans* reverse photoisomerization was considered. Both thermal treatment (T = 80°C) and blue light stimulation (λ = 445 nm, 440 mW) were tested, showing complete recovery of the *trans* isomer after 10 minutes of stimulation. However, by the acquisition of UV spectra at different time points, a different kinetic recovery was observed. When blue light was used, an almost complete recovery of the *trans* isomer immediately after 1 minutes was observed. On the other hand, when the reverse isomerization was thermally promoted, a more gradual recovery was noted.

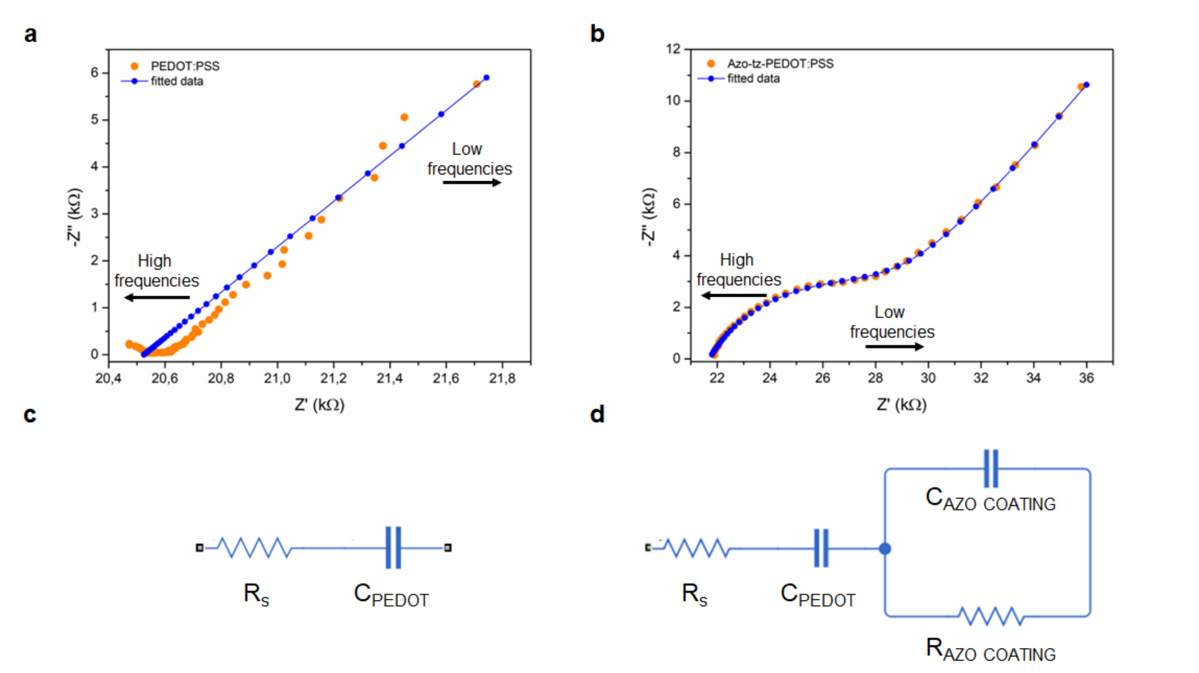

Supplementary Figure 12. EIS fitting and equivalent circuits. Nyquist plots with fitted data (blue lines) of a) PEDOT:PSS and b) azo-tz-PEDOT:PSS. Equivalent circuit obtained by fitted data of c) PEDOT:PSS and d) azo-tz-PEDOT:PSS**.** Electrochemical impedance spectroscopy (EIS) analysis of azo-tz-PEDOT:PSS was carried out. Bare PEDOT:PSS and azo-tz-PEDOT:PSS were characterized by two different equivalent circuits. Bare PEDOT:PSS behavior was described and fit through a series of resistance and capacitance[10]. The former considers contact and electrolyte resistance, while the latter represents the film capacitance. Despite its simplicity, this model can adequately fit the measurements describing the switch between the high-frequency resistive behavior and the low-frequency capacitive-dominated behavior. Furthermore, a constant phase element (CPE) was used instead of an ideal capacitance to include all the non-idealities typical of conductive polymer thin films[11]. In contrast, azo-tz-PEDOT:PSS low-frequency data can be fitted using a semicircular shape representing the charge transfer and the barrier properties of the surface[12]. Resistance and capacitance (CPE) connected in parallel, commonly used to model physical and chemical surface coatings[13,14], were added to the previously shown circuit. These new circuital elements model the leaky barrier behavior in which the capacitance describes the accumulation of ions at the interface, while the charge transfer resistance reflects the charge transfer at the conductive polymer/electrolyte interface.

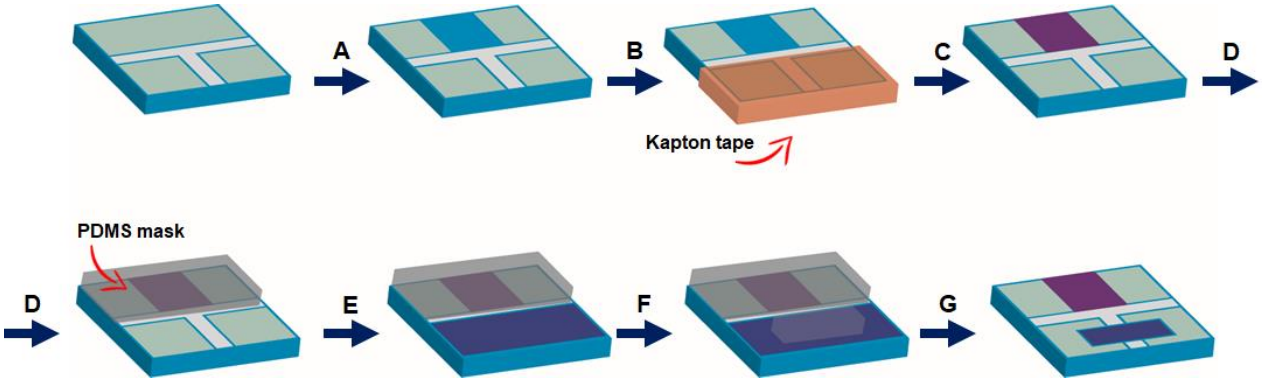

Supplementary Figure 13. OPECT fabrication process. A) N_3-PEDOT:PSS electrodeposition (blue square), B) isolation of ITO square electrodes with Kapton tape, C) functionalization of the polymer with azo-alkyne to give azo-tz-PEDOT:PSS (violet square), D) coverage of the azo-tz-PEDOT:PSS film with a rectangular PDMS mask (gray) and O_2 plasma activation, E) spin coating of PEDOT:PSS solution, F) isolation of the desired part of sPEDOT:PSS corresponding to the OPECT channel G) O_2 plasma etching to afford the desired dimension and geometry of sPEDOT:PSS channel. The fabrication of the OPECT bearing the azo-tz-PEDOT:PSS as a planar gate involved 7 steps. Patterned ITO substrates were previously cleaned in an ultrasonic bath for 10 minutes in each of the following solvents: Alconox® detergent solution, DI water, acetone, and 2-propanol. Then, N_3-PEDOT:PSS was electrodeposited on the upper part of the ITO-coated glass and functionalized following the previously described *click chemistry* reaction procedure (Methods), insulating first the bottom part of ITO with Kapton tape. The azo-tz-PEDOT:PSS gate was then covered with a PDMS mask, and the bottom part of the ITO was activated through O_2 plasma treatment (20 W, 2 minutes) to allow spin coating of the PEDOT:PSS (sPEDOT:PSS) channel. The sPEDOT:PSS layer was then O_2 plasma etched (100 W, 15 minutes) through a PDMS hard mask to obtain the desired channel geometry and dimension.

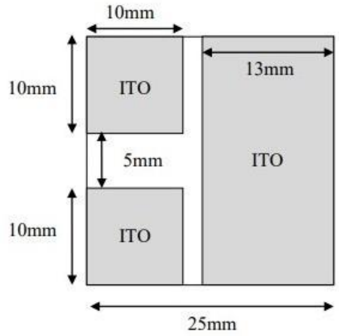

Supplementary Figure 14. ITO substrate Layout. Customized patterned ITO-coated glass used for the fabrication of the azo-OPECT.

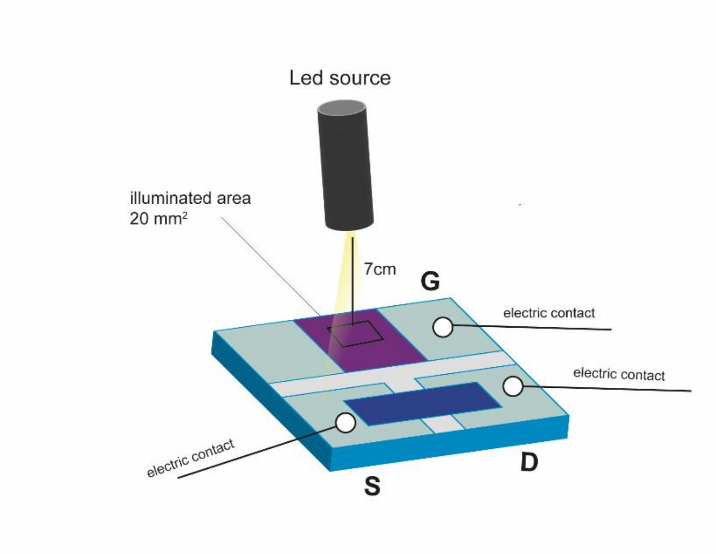

Supplementary Figure 15. Schematics of the optoelectronic measurement setup. Setup used for OPECT measurements under light conditions.

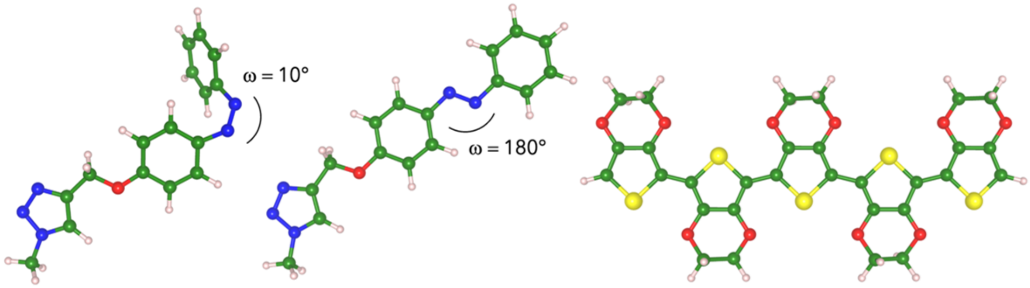

	cis azo-tz	trans azo-tz	PEDOT
λ_{max} (nm)	485	349	507
f (arb. units)	0.056	1.006	1.703
orbitals	HOMO → LUMO	HOMO → LUMO	HOMO → LUMO

Supplementary Figure 16. Structural models of *cis* azo-tz, *trans* azo-tz, and PEDOT building blocks. The C-N-N-C dihedral values, ω, are shown to highlight the *cis* and *trans* conformers. TDDFT-derived electron transitions are reported in the bottom panel with related details on the wavelength values, oscillator strengths and the type of orbitals involved. The structural models for the azo-tz-PEDOT systems consist of two building blocks, the azobenzene-triazine (azo-tz) moiety and the poly(3,4-ethylenedioxythiophene) (PEDOT) moiety. The former was considered in both the *cis* and *trans* conformations (*i.e.*, along the C-N-N-C dihedral, that is, the N=N bond in the azobenzene), while the latter is made of 5 monomers. The *cis*-*trans* energy difference computed at the B3LYP/6-31G(d,p) level of theory is $DE_{(cis-trans)}$ = 0.691 eV (15.936 kcal/mol). By deriving the electron transitions for each structure, we could validate our method and confirm the reliability of our model (Results and Discussion, Figure 1c for comparison).

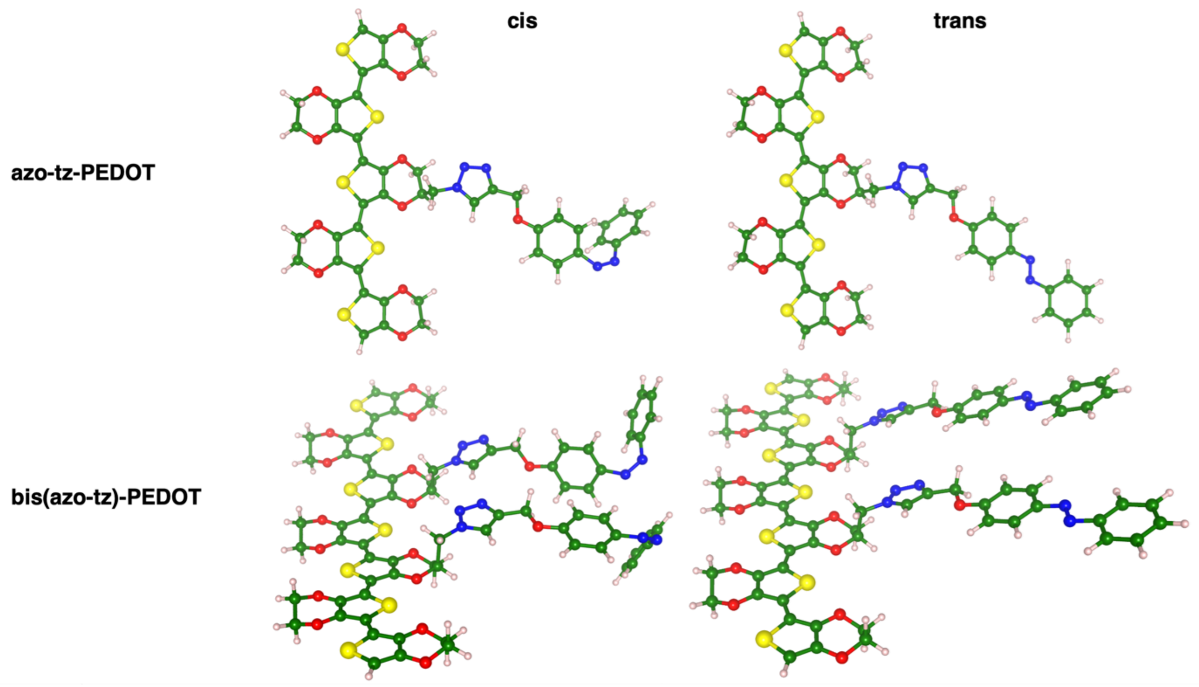

Supplementary Figure 17. Ground-state structures of *cis* and *trans* azo-tz-PEDOT systems. Ground-state structures of *cis* and *trans* azo-tz-PEDOT systems obtained at the B3LYP/6-31G(d,p) level of theory obtained at the B3LYP/6-31G(d,p) level of theory. Bis(azo-tz)-PEDOT was also considered to model higher coverage.

The azo-tz-PEDOT systems were modeled by considering either a mono- or a bi-substitution of the azo-tz moiety on the PEDOT backbone. The former was labeled bis(azo-tz-PEDOT) and requires a longer 7-monomer PEDOT chain. Nevertheless, *cis* and *trans* conformers were considered, and the related energy difference was not altered (~ 0.68-0.69 eV), suggesting that the linkage to the PEDOT backbone does not affect the conformational freedom along the azobenzene chain.

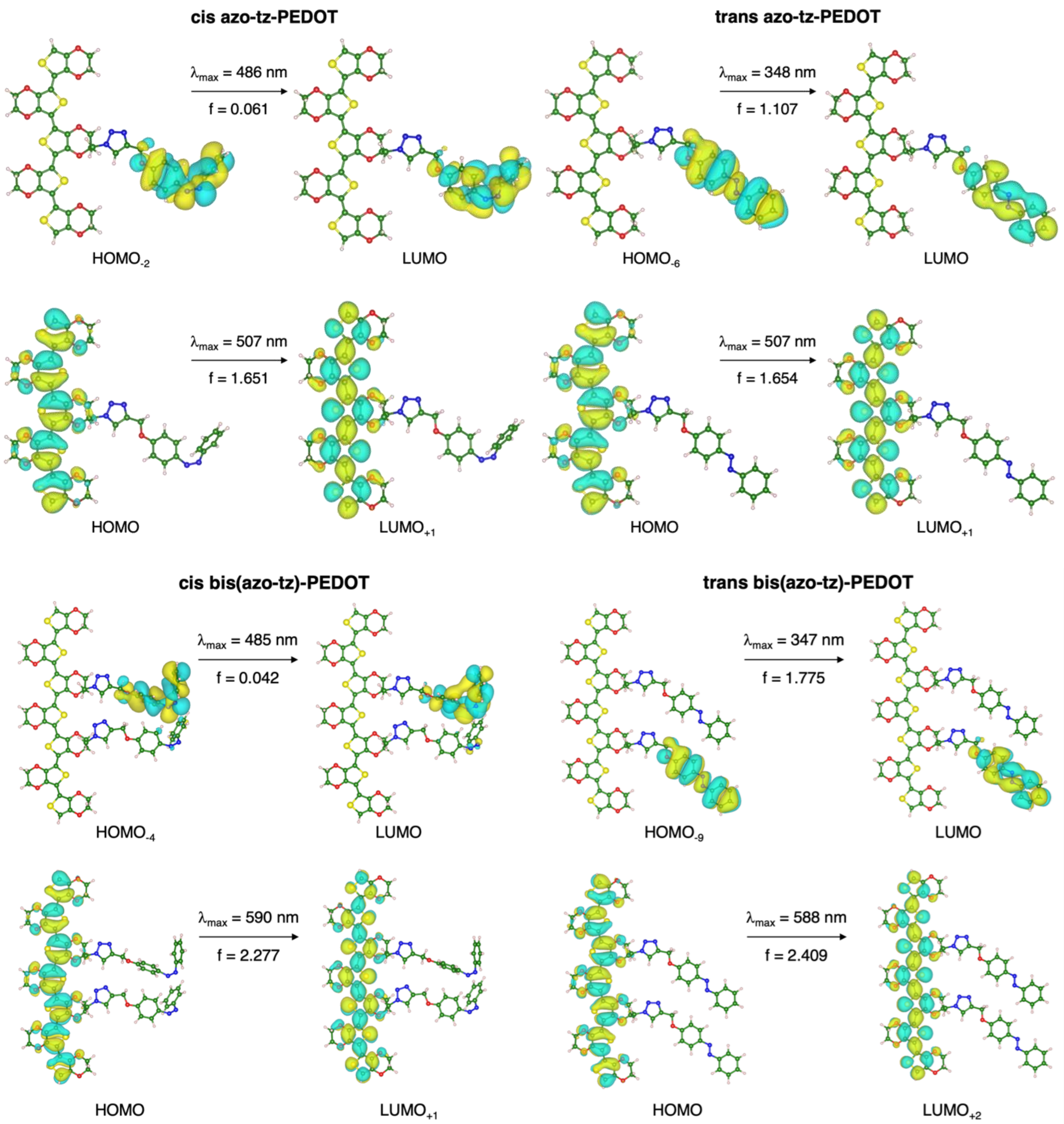

Supplementary Figure 18. Electron transitions computed with TDDFT calculations. Electron transitions computed with TDDFT calculations for (from left to right) *cis* and *trans* (from top to bottom) mono- and bis(azo-tz)-PEDOT systems. The optical properties of *cis*/*trans* mono/bis azo-tz-PEDOT were determined from TDDFT calculations. The vertical excitations, together with wavelengths and oscillator strengths, while the involved orbitals are depicted on the chemical structure. Here, both mono- and bi-substituted models can reproduce the typical transition at ~ 348 nm owing to *trans* azobenzene, as well as the less intense excitation at ~ 485 nm on account of the *cis* conformer.

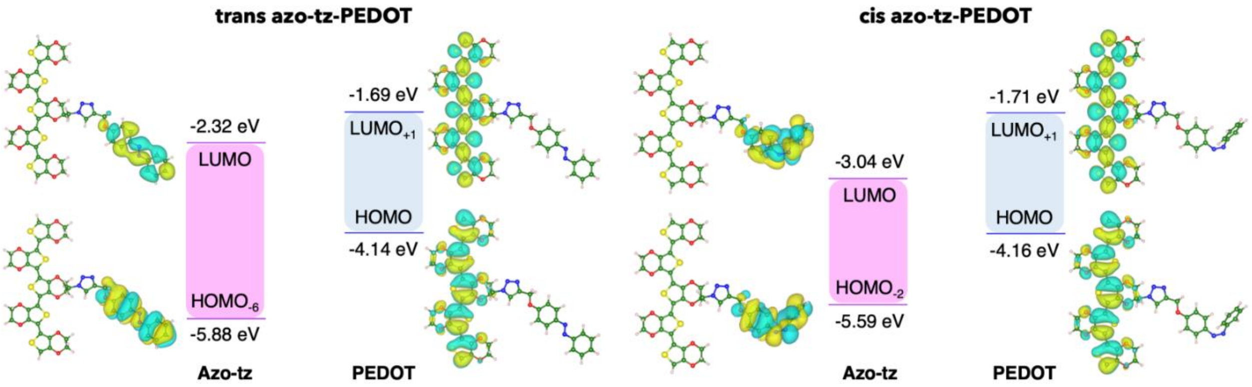

Supplementary Figure 19. Energy levels of trans and cis azo-tz-PEDOT. Energy levels of trans (left) and cis (right) azo-tz-PEDOT obtained from DFT-TDDFT calculations: HOMO/LUMO orbitals from azobenzene and PEDOT moieties are highlighted in pink and blue, respectively. The values are reported in electronvolts. Corresponding density surfaces plotted on the minimum-energy structures are also displayed to the side (isosurface level 0.01 eV/Å3). The energy levels alignment obtained for the *cis* and *trans* conformers of azo-tz-PEDOT systems is similar, suggesting that the conformational orientation of azobenzene does not alter the underlying energetics; thus, *trans-cis* isomerization is not expected to be a key process in the whole charge transfer process. From the results emerges that MO energies and localizations for cis and *trans* isomers are both suitable for direct injection for a photoexcited electron from the LUMO level of the azobenzenes to the HOMO level of PEDOT. This means that even considering a not complete conversion from *trans/cis* isomer at the gate, the energy diagram of frontier orbitals is not qualitatively affected by the different isomer conformations.

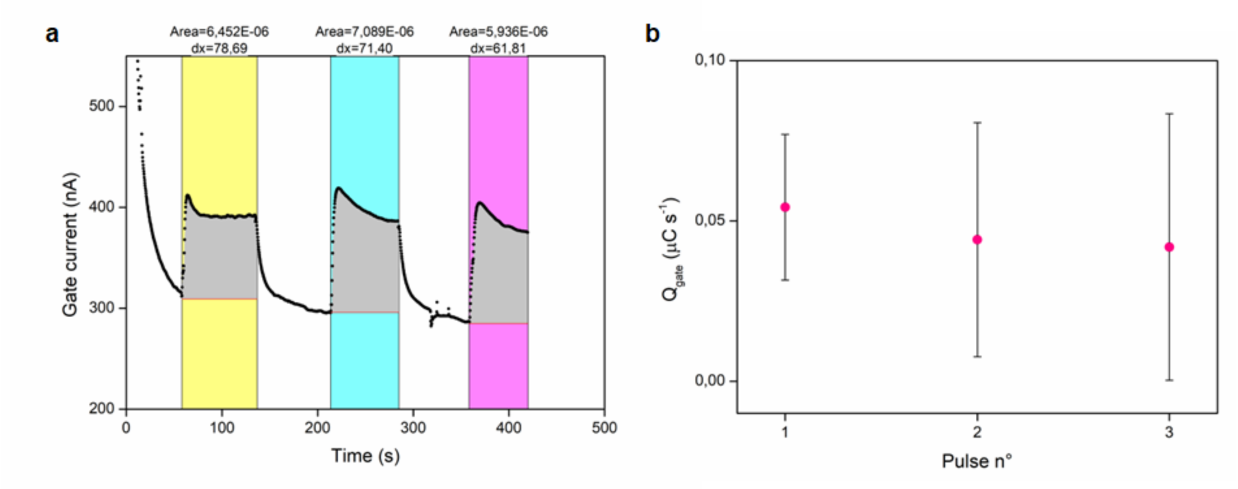

Supplementary Figure 20. Calculation of the accumulated charge on azo-tz-PEDOT:PSS gate electrode. a) Schematics of the calculation of gate charge by integration of the gate current (I_{gs}) over time during UV irradiation of the azo-tz-PEDOT:PSS planar gate. Gray shaded portions are related to the integrated area; b) graph of normalized gate charge *vs* pulse number. The pulsed measurements with UV light stimulation were performed by fixing the V_{ds} and the V_{gs} at -200 mV and 0 mV, respectively, and by exposing the gate electrode to 60 seconds in the dark and 60 seconds of the highest intensity of UV light (λ = 365 nm, 2.8 W/cm^2). When the light stimulus was applied, a rapid decrease in I_{ds} was observed, which underwent a rapid increase when the stimulus was removed, suggesting the accumulation of charge on the gate electrode surface. Thus, the amount of gathered charge was calculated by integrating the gate current during the illumination time for each light pulse and then normalized with respect to the illumination time. The accumulated charge on the gate electrode was calculated and found to be constant for the first three pulses (Numerical Values: 0.054 ± 0.023 µC/s, 0.044 ± 0.036 µC/s and 0.042 ± 0.042 µC/s, N = 5). Additionally, the accumulated charge was calculated only for the first pulse (t = 60 s, λ = 365 nm) while varying the light intensity (20% = 0.56 W/cm^2, 60% = 1.7 W/cm^2, 100% = 2.8 W/cm^2). The results showed a considerable increase in the charge accumulation from 0.56 W/cm^2 to 1.7 W/cm^2 (Numerical Values: 0.020 ± 0.012 µC/s and 0.058 ± 0.040 µC/s, respectively; N = 3), which stabilizes with increasing light intensity up to 2.8 W/cm^2 (Numerical Value: 0.051 ± 0.022 µC/s; N = 3). This result confirms that the current variation was mainly dominated by the photoinduced charge transfer mechanism, which was proportional to the light intensity (Results and Discussion, Figure 2d).

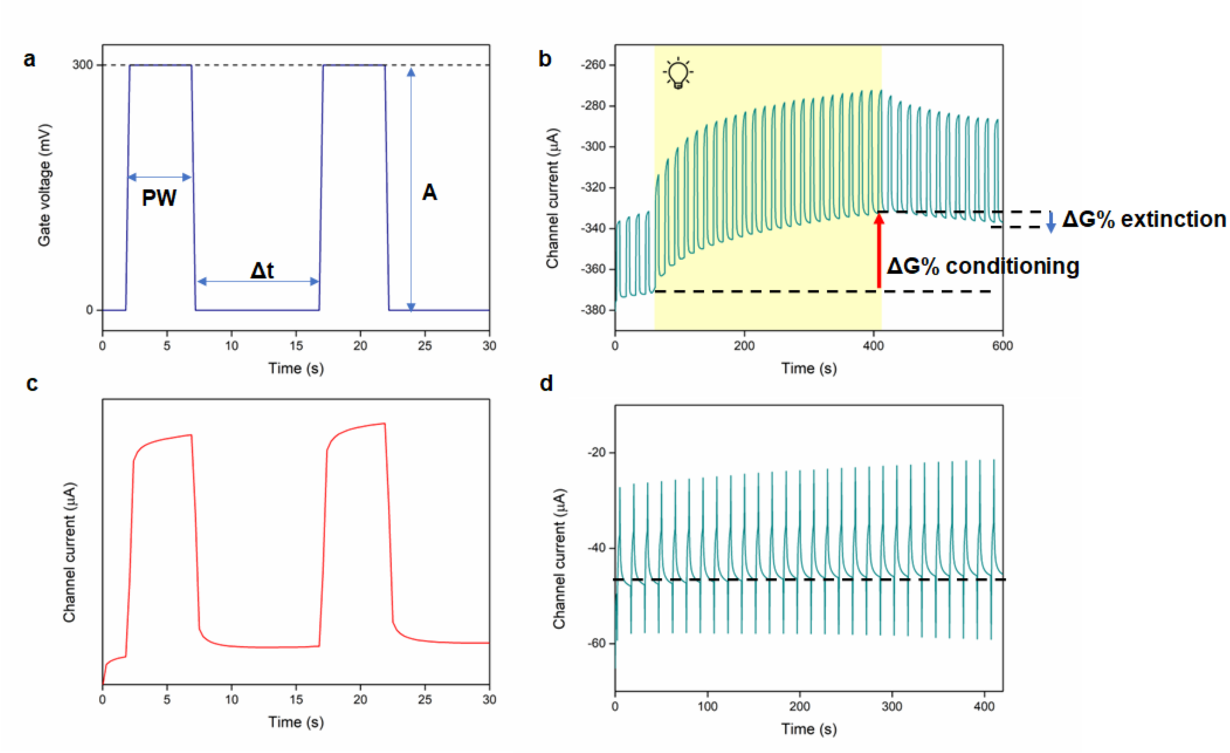

Supplementary Figure 21. Operation of the azo-OPECT by applying square voltage pulses under UV-light. a) Square voltage pulsed input applied at the gate electrode (blue solid line), showing the pulse amplitude (A or V_{gs}), duration of the pulse (PW) and delay between pulses (Δt); b) graph of the channel current recorded during the measurement: when the gate electrode was exposed to UV light (yellow box), the conductance of the device largely increased after 6 minutes of illumination; after turning the UV lamp off, the conductance slowly decreased in the dark for 3 minutes; c) channel current response recorded after the application of a voltage pulsed input (red solid line); d) graph of the channel current recorded during the measurement in which the PW was 3 seconds in dark conditions: no difference in channel current was observed. The I_{ds} of the OPECT was monitored during the application of positive voltage pulses, keeping the channel voltage constant at V_{ds} = -200 mV. During this experiment, the gate pulse was characterized by keeping a constant voltage amplitude (A) V_{gs} = 300 mV, pulse width (PW), and time interval between consecutive pulses (Δt). The total duration of the pulse was maintained at 15 seconds and the resulting channel current was recorded. The same measurement was also performed by applying pulse voltages at the azo-tz-PEDOT:PSS gate with a PW = 3 seconds in dark conditions. Here, the recorded channel current remained constant, demonstrating that no conductance variation can be observed without light stimulation.

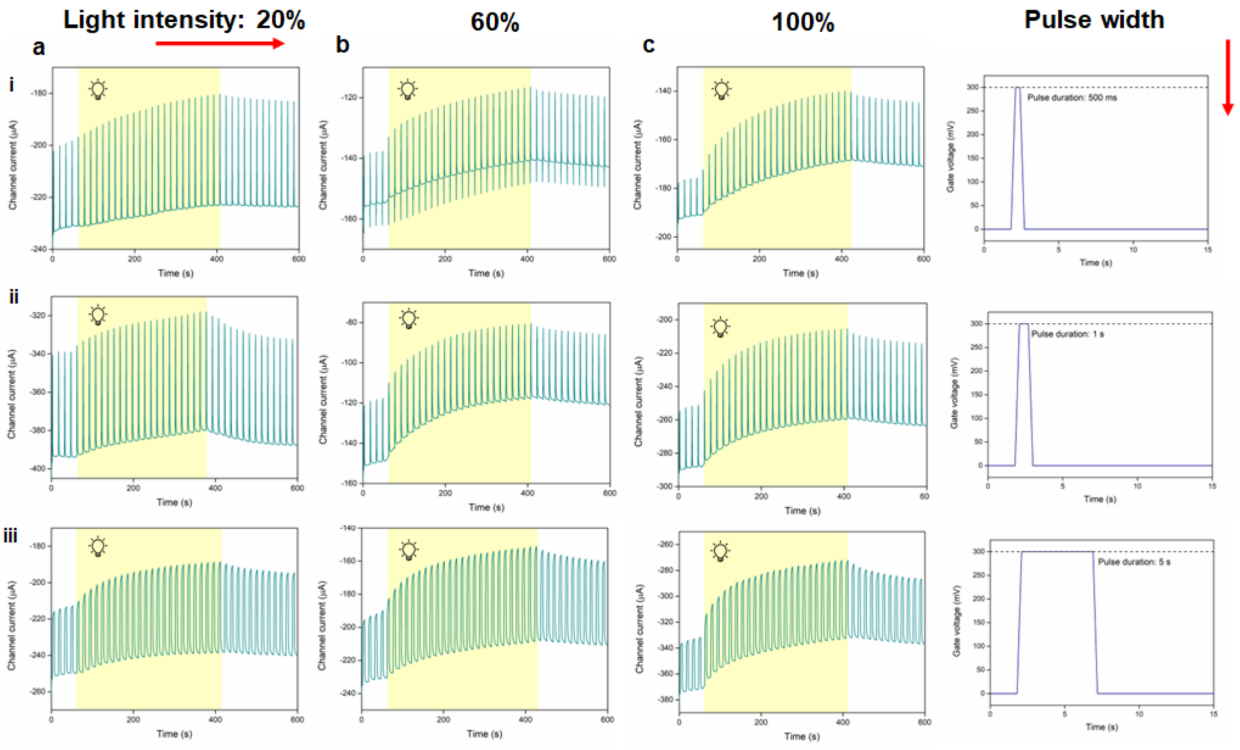

Supplementary Figure 22. Operation of the azo-OPECT under UV-light stimulation with different light intensity and pulse width. Graphs of the channel current has been recorded during the measurement, first fixing the PW while changing the light intensity of the UV light (yellow box) a) 20% b) 60% c) 100% (λ = 365 nm, 0.56 W/cm^2, 1.7 W/cm^2, 2.8 W/cm^2, t = 6 minutes). Then, fixing the light intensity and varying the PW applied to the gate electrode i) 500 ms, ii) 1 s and iii) 5 s. In both cases, the conductance (referred to as $g = -\partial I_{ds}/\partial V_{ds}$) of the device largely increased after 6 minutes of illumination (conditioning), while after the UV lamp was turned off and the samples were kept in the dark for 3 minutes, the conductance slowly decreased (extinction). The conductance was calculated during the first and last pulse currents in dark and lighting conditions, respectively.

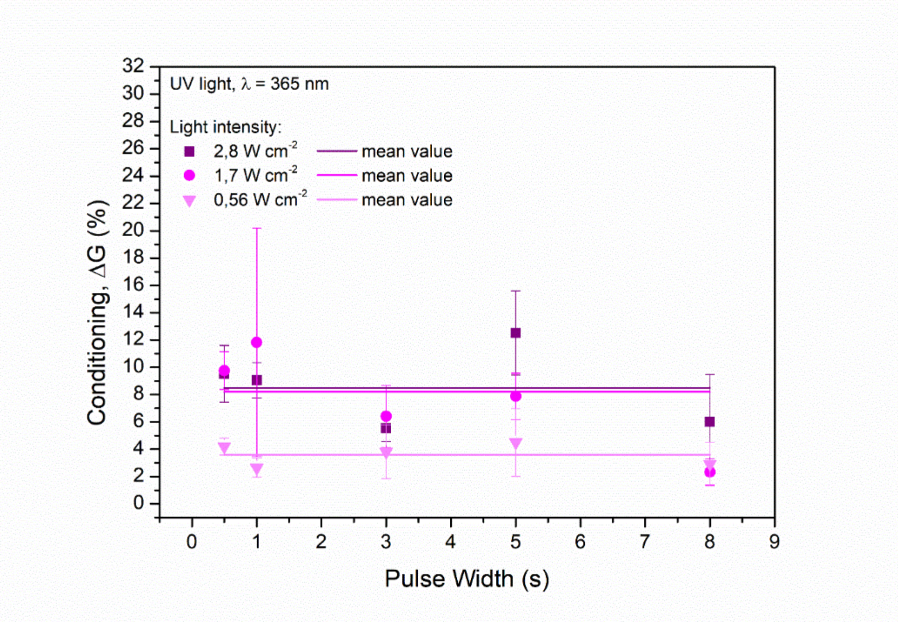

Supplementary Figure 23. Conductance variation *vs.* pulse width. The data reveal that there is no dependence between the conditioning and PW. The percentage conductance variation (ΔG%) was also evaluated, and each value and standard deviations are reported in the Supplementary Table 2 and 3. When keeping the light intensity constant while changing the PW, no considerable conductance difference was found, demonstrating that the light effect was independent of the duration of the electrical pulse.

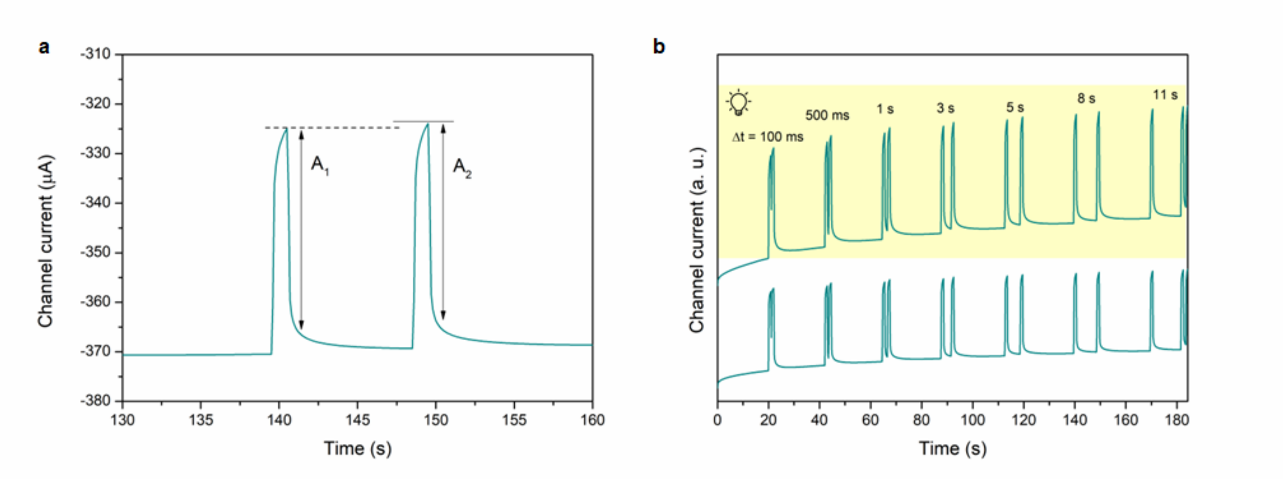

Supplementary Figure 24. PPF index calculation. a) Schematic of PPF synaptic behavior in which two consecutive current peaks were recorded by applying two identical pulse voltages with an interval of Δt = 8 s to the gate electrode; b) example of recorded channel spikes by applying pulse voltages with V_{gs} = +300 mV, PW = 1 second and Δt = 100 ms, 500 ms, 1 s, 3 s, 5s , 8 s, 11 s at the gate electrode in dark and UV-light conditions (yellow box). Paired-pulse facilitation (PPF) is a type of synaptic behavior that can usually be emulated in OPECTs by applying two identical pulse voltages with short intervals (Δt) to the gate electrode. This causes two consecutive current peaks in the postsynaptic current (channel current) in which the amplitude of the second spike (A_2) was higher than the amplitude of the first spike (A_1) when the second pulse voltage was close enough to the first one. During the measurement, pulse voltages of different durations were applied to the gate electrode in dark and UV light conditions (λ = 365 nm, 2.8 W/cm^2). The PPF index was calculated as the percentage ratio of A_2/A_1 and was reported *vs* Δt, indicating an interval between 5 s and 8 s as the optimal value to shift from STP to LTP behavior by applying both electrical and optical stimuli (Results and Discussion, Figure 3 e). The PPF index was computed using N=3 devices. Numerical data are available in the Supplementary Table 4.

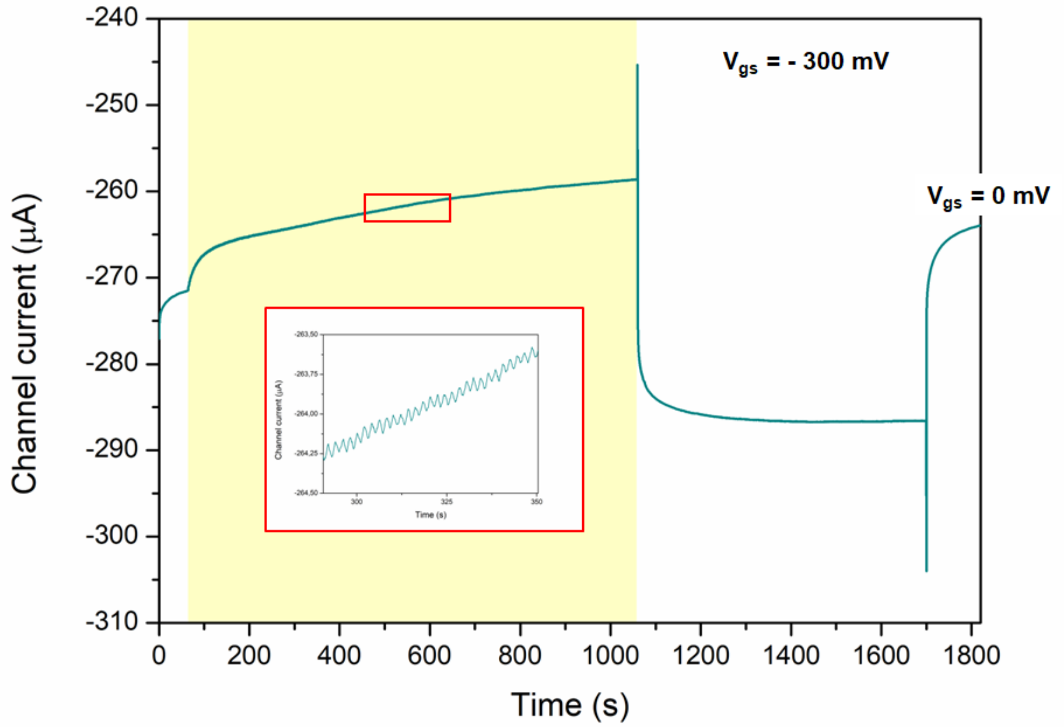

Supplementary Figure 25. Writable/erasable optoelectronic memory by applying 500 light pulses. Channel current recorded during the application of 500 light pulses (λ = 365 nm, 2.8 W/cm^2 of light intensity, PW = 1 s, Δt = 1 s) (yellow box) and a negative voltages V_{gs} = -300 mV (total duration 10 minutes). Finally, the electrical stimulus was removed and kept at 0 mV until the end of the measurement. During this measurement, 500 light pulses (PW = 1 s, Δt = 1 s) were applied to the azo-tz-PEDOT:PSS gate, decreasing the channel current and causing 500 discrete and stable changes in conductance (see inset). After the illumination of the sample, a negative voltage of V_{gs} = - 300 mV (total duration 10 minutes) was applied, increasing again the channel current. Finally, by removing the electrical stimulus, the current values decreased again bringing the synaptic conditioning back to the initial values.

These results suggested that by applying a negative voltage to the gate, a backflow of cations is facilitated from the channel to the electrolyte and induces a reoxidation effect of PEDOT:PSS, as also previously reported[15].

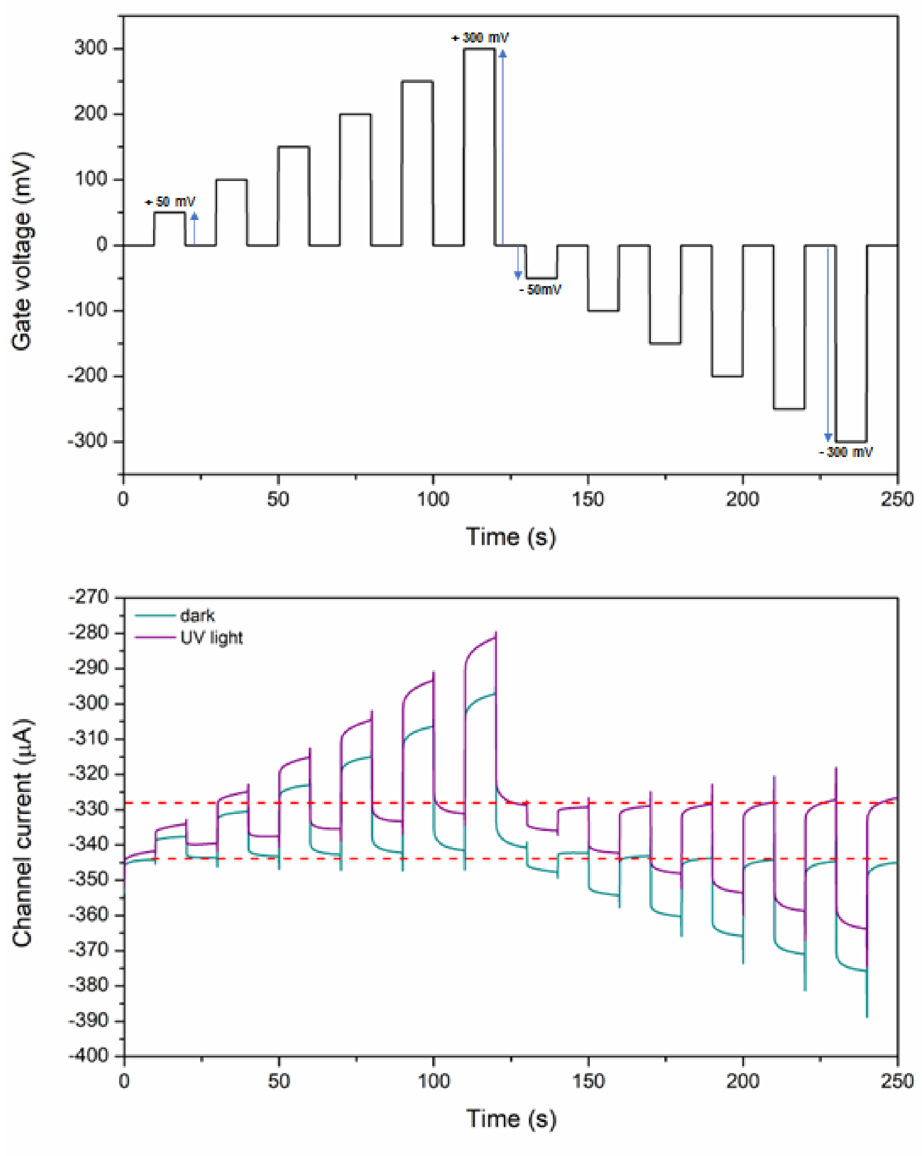

Supplementary Figure 26. Control measurements on bare PEDOT:PSS. Channel current recorded after applying a train of positive (V_{gs} from +50 mV to +300 mV, PW = 60 s and Δt = 10 s) and negative (V_{gs} from -50 mV to -300 mV, PW = 60 s and Δt = 10 s) electrical pulses both in dark (cyan solid line) and UV light (violet solid line) conditions (λ = 365 nm, 2.8 W/cm^2) on an OECT bearing a gate made of bare PEDOT:PSS.

Control measurements were performed using also an OPECT bearing a gate made of bare PEDOT:PSS by applying both positive square pulse voltages (V_{gs} from +50 mV to +300 mV, PW = 60 s and Δt = 10 s) and negative square pulses (V_{gs} from -50 mV to -300 mV, PW = 60 s and Δt = 10 s) under dark and light conditions. Any modification in the channel current was observed during the application of negative pulses, confirming that azobenzene moieties are involved in the charge transfer process.

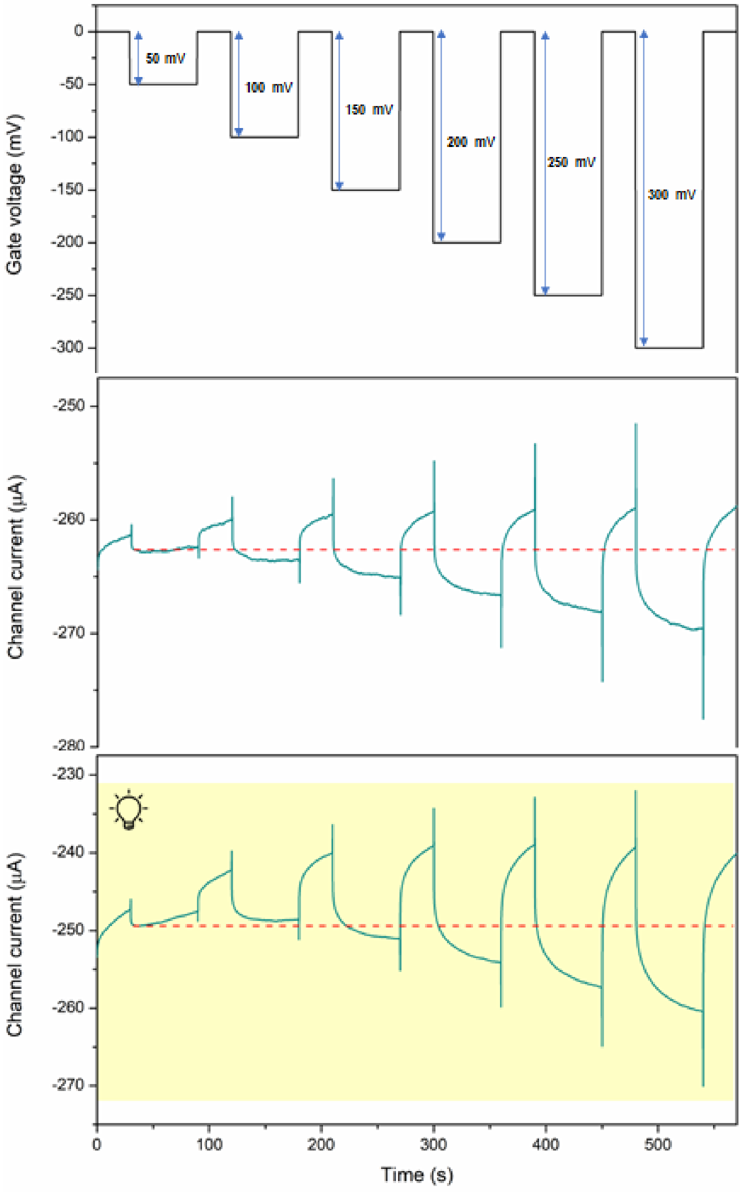

Supplementary Figure 27. Competition between the effect induced by light and electric pulses. Channel current recorded after applying a train of pulse voltages with a PW = 60 s, Δt = 30 s and amplitude between V_{gs} = -50 mV to V_{gs} = -300 mV (with a step of -50 mV) under dark and UV light conditions (λ = 365 nm, 2.8 W/cm^2, yellow box): the application of negative voltages to the gate competes with UV irradiation; turning on the UV lamp decreased the channel current, reaching a constant value when an electric pulse of -100 mV was applied. With the application of a negative pulse voltage, a current increase was observed again. A series of square pulse voltages was applied at the gate electrode under both dark and UV-light conditions. In dark conditions, the channel current increased with increasing voltage amplitude. Performing the same measurement under UV light conditions, competition between the effect induced by light and electric pulses was observed during the first three pulses. Indeed, after turning the UV lamp on, the channel current decreased until reaching a constant value when an electric pulse of -100 mV was applied.

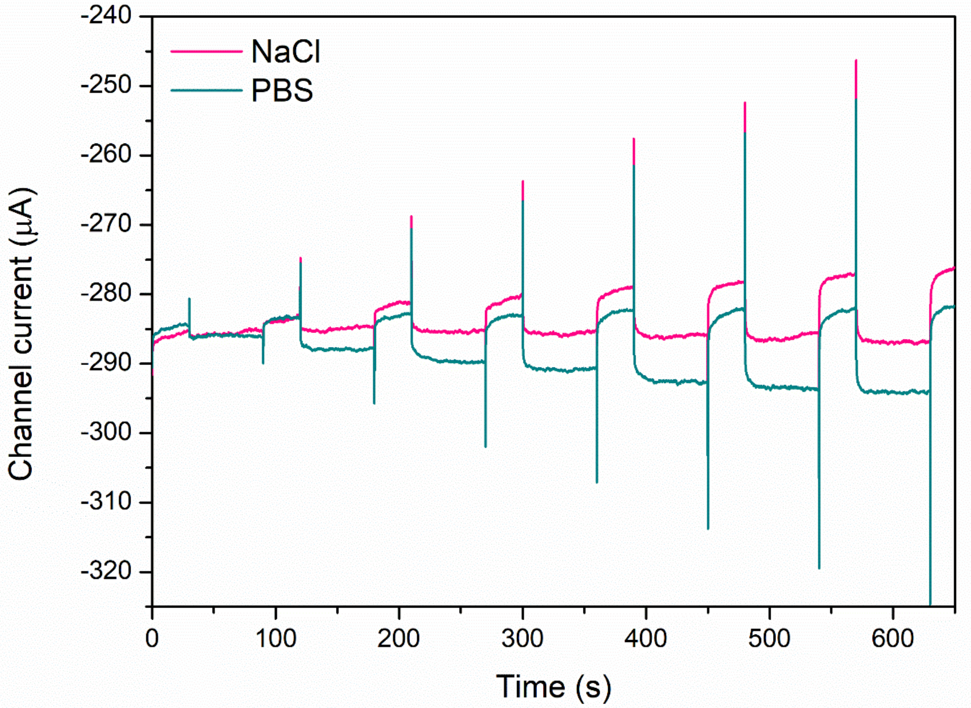

Supplementary Figure 28. Role of cations involved in the optoelectronic mechanism. Channel current recorded after applying a train of pulse voltages with a PW = 60 seconds and amplitude between V_{gs} = -50 mV to V_{gs} = -350 mV (with a step of -50 mV) under dark conditions by using PBS (cyan solid line) and NaCl 100 mM (pink solid line) as the electrolyte. To better understand the role of cations involved in the reduction of azo-tz-PEDOT:PSS, the PBS electrolyte was replaced with an aqueous solution of NaCl 100 mM. By applying a train of negative square voltages to the gate electrode, no increase in the channel current was observed. Such behavior suggests that protons are involved in the reduction mechanism since a NaCl solution has less species able to donate H^+ with respect to KH_2PO_4 and Na_2HPO_4 which are indeed present in the PBS electrolyte: the H^+ molar concentration in the PBS solution was higher than NaCl 100 mM solution by approximately two orders of magnitude (10^{-2} mM and 10^{-4} mM for PBS solution and NaCl solution, respectively).

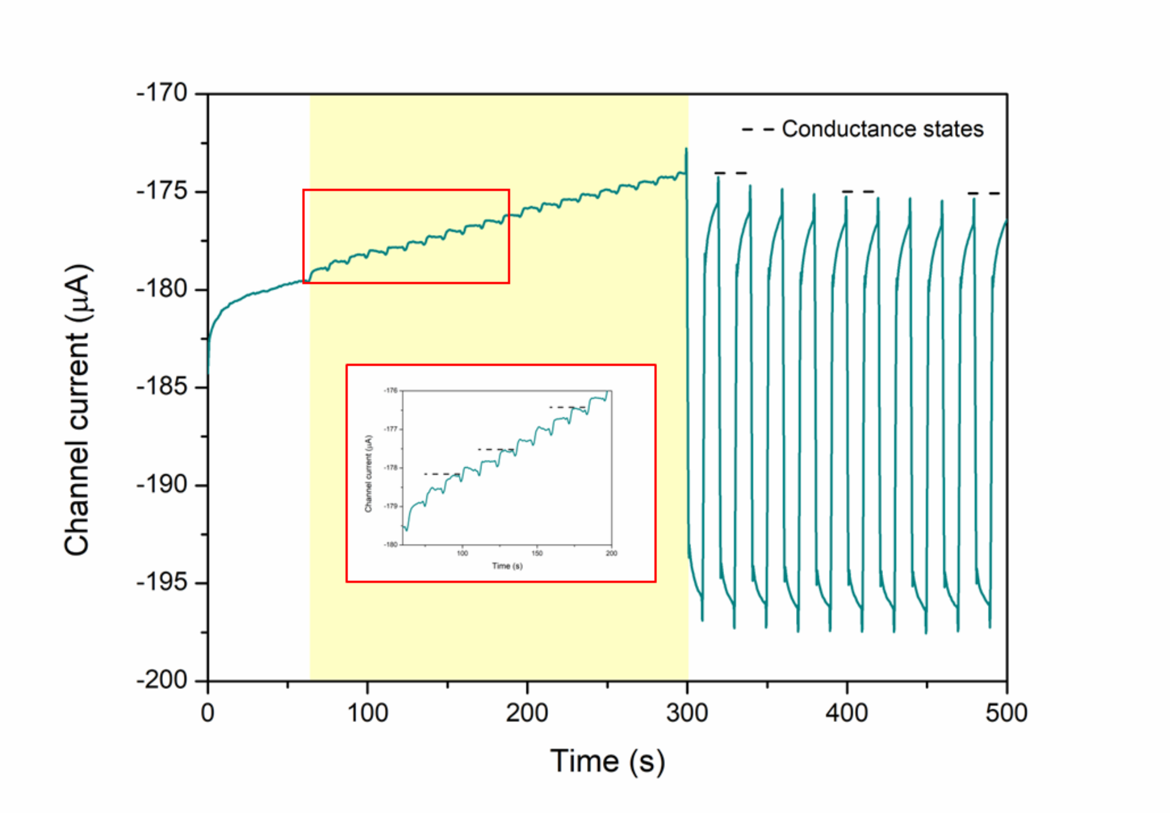

Supplementary Figure 29. Writable/erasable optoelectronic memory by applying 20 light pulses. Channel current recorded during the application of 20 light pulses (λ = 365 nm, 2.8 W/cm^2 of light intensity, PW = 10 s, Δt = 10 s) (yellow box) and 10 negative pulse voltages with PW = 2 s, Δt = 10 s, V_{gs} = -300 mV. the writable/erasable behavior of the optoelectronic memory is reported. During the measurement, 20 light pulses were applied to the azo-tz-PEDOT:PSS gate, causing a decrease in the channel current and a consequent change in conductance in discrete and stable states (inset, black dashed line). After light irradiation of the sample, a train of 10 negative pulse voltages was applied, increasing the channel current and modulating the conductance in discrete and stable states (black dashed line). Such writable (conditioning) and erasable (extinction) behavior demonstrates the possibility of using azo-OPECT as optoelectronic memory, in which information can be stored through light pulses and cancelled by applying electric stimuli.

Supplementary Tables

	R_s [kΩ]	C_{pedot} [µF]	N_{pedot}	$R_{azo\ coating}$ [kΩ]	$C_{azo\ coating}$ [µF]	$N_{azo\ coating}$
PEDOT:PSS	6.99 ± 0.17	490 ± 14	0.51 ± 0.02	-	-	-
PEDOT:PSS after UV light	7.71 ± 0.10	452 ± 20	0.5 ± 0.03	-	-	-
Azo-tz-PEDOT:PSS	9.78 ± 1.15	80.5 ± 19.3	0.55 ± 0.03	2.05 ± 0.55	3.63 ± 0.85	1.03 ± 0.02
Azo-tz-PEDOT:PSS after UV light	8.89 ± 1.16	52.3 ± 15.0	0.64 ± 0.03	2.48 ± 1.43	4.73 ± 2.02	0.94 ± 0.07

Supplementary Table 1. Numerical data of EIS measurements obtained through data fitting. Data were compared to pristine PEDOT:PSS electrodes prior to (top raw) and after the chemical functionalization (bottom raw) (PEDOT:PSS N = 3, Azo-tz-PEDOT:PSS N = 4).

Light intensity	ΔG% conditioning (6 minutes)				
	PW: 500 ms	PW: 1 s	PW: 3 s	PW: 5 s	PW: 8 s
100%	9.5 ± 2.1	9.1 ± 1.3	5.5 ± 1.0	12.5 ± 3.1	6.0 ± 3.5
60%	12.5 ± 3.1	11.8 ± 8.4	6.4 ± 2.3	7.9 ± 1.7	2.3 ± 1.0
20%	4.2 ± 0.6	2.7 ± 0.7	3.8 ± 1.9	4.5 ± 2.5	2.9 ± 1.6

Supplementary Table 2. Synaptic conditioning. Conditioning calculated as percentage conductance variation (ΔG%) by changing the light intensity of the UV light (20%, 60%, 100%, N = 3 of each) and the PW applied to the gate electrode (500 ms, 1 s, 3 s, 5 s and 8 s, N = 3 of each).

Light intensity	ΔG% extinction (3 minutes)				
	PW: 500 ms	PW: 1 s	PW: 3 s	PW: 5 s	PW: 8 s
100%	-0.8 ± 0.9	-0.9 ± 1.2	-0.6 ± 0.2	-2.7 ± 3.1	-0.2 ± 1.4
60%	-0.7 ± 1.1	-2.0 ± 1.3	-0.6 ± 0.2	-0.7 ± 0.6	0.2 ± 0.3
20%	0.9 ± 1.0	-0.1 ± 1.6	-0.1 ± 0.6	-0.2 ± 0.6	0.9 ± 1.0

Supplementary Table 3. Synaptic extinction. Extinction calculated as percentage conductance variation (ΔG%) by changing the light intensity of the UV light (20%, 60%, 100%, N = 3 of each) and the PW applied to the gate electrode (500 ms, 1 s, 3 s, 5 s and 8 s, N = 3 of each).

Time interval Δt (s)	PPF (%) Device #1, dark	PPF (%) Device #1, light	PPF (%) Device #2, dark	PPF (%) Device #2, light	PPF (%) Device #3, dark	PPF (%) Device #3, light
0.1	98.9	98.3	99.6	99.1	99.9	99.5
0.5	99.1	98.4	99.4	99.1	99.9	99.5
1	99.5	98.9	99.7	99.4	99.9	99.7
3	99.7	99.4	99.8	99.6	99.9	99.8
5	99.7	99.3	99.8	99.5	99.9	99.7
8	100.0	100.0	100.0	100.0	100.0	100.0
11	100.0	100.0	100.0	100.0	100.0	100.0

Supplementary Table 4. PPF index of 3 devices computed in both light and dark conditions.

Supplementary References

1.Scavetta, E. *et al.* Dopamine amperometric detection at a ferrocene clicked PEDOT:PSS coated electrode. *J. Mater. Chem. B* **2**, 2861–2867 (2014).

2. Casas-solvas, J., Martos-Maldonado, M. & Vargas-Berenguel, A. Synthesis of β-cyclodextrin derivatives functionalized with azobenzene. *Tetrahedron* **64**, 10919–10923 (2008).

3. Castagnola, V., Bayon, C., Descamps, E. & Bergaud, C. Morphology and conductivity of PEDOT layers produced by different electrochemical routes. *Synth. Met.* **189**, 7–16 (2014).

4.Bu, H.-B. *et al.* "Click"-functionalization of conducting poly(3,4-ethylenedioxythiophene) (PEDOT). *Chem. Commun.* **11**, 1320–1322 (2008).

5.Greczynski, G. *et al.* Photoelectron spectroscopy of thin films of PEDOT–PSS conjugated polymer blend: a mini-review and some new results. *J. Electron Spectrosc. Relat. Phenom.* **121**, 1–17 (2001).

6. Jin Bae, E., Hun Kang, Y., Jang, KS. & Yun Cho, S. Enhancement of thermoelectric properties of PEDOT:PSS and tellurium-PEDOT:PSS hybrid composites by simple chemical treatment. *Sci. Rep.* **6**, 18805 (2016)

7.Mengistie, D. A., Ibrahem, M. A., Wang, P.-C. & Chu, C.-W. Highly conductive PEDOT:PSS treated with formic acid for ITO-free polymer solar cells. *ACS Appl. Mater. Interfaces* **6**, 2292–2299 (2014).

8.Rodríguez-Jiménez, S. *et al.* Electroactive metal complexes covalently attached to conductive PEDOT films: a spectroelectrochemical study. *ACS Appl. Mater. Interfaces* **13**, 1301–1313 (2021).

9.Wu, B., Cao, B., Taylor, I. M., Woeppel, K. & Cui, X. T. Facile synthesis of a 3,4-ethylene-dioxythiophene (EDOT) derivative for ease of bio-functionalization of the conducting polymer PEDOT. *Front. Chem.* **7**, 178 (2019).

10. Koutsouras D. A. *et al*. Impedance spectroscopy of spin-cast and electrochemically deposited PEDOT:PSS films on microfabricated electrodes with various areas. *ChemElectroChem*. **4**, 2321-2327 (2017).

11.Inzelt, G. & Láng, G. G. *Electropolymerization: Concepts, Materials and Applications Ch. 3*, 51–76 (Wiley-VCH Verlag GmbH & Co. KGaA, Germany, 2010).

12. Vivier, V. & Orazem, M. E. Impedance analysis of electrochemical systems, *Chem. Rev*. **122**, 11131-11168 (2022).

13.Tano de la Hoz, M. F. *et al.* RGD-functionalization of anodized zirconium as a potential strategy for biomedical application: an in vitro study. *Surf. Coat. Technol.* **433**, 128088 (2022).

14.Khajavian, E. *et al.* Tuning surface wettability of aluminum surface and its correlation with short and long term corrosion resistance in saline solutions. *Surf. Coat. Technol.* **429**, 127950 (2022).

15.van de Burgt, Y. *et al.* A nonvolatile organic electrochemical device as a low-voltage artificial synapse for neuromorphic computing. *Nat. Mater.* **16**, 414–418 (2017).
